# Toddlers Prefer Agents Who Help Those Facing Harder Tasks

**DOI:** 10.1162/opmi_a_00129

**Published:** 2024-04-10

**Authors:** Brandon M. Woo, Shari Liu, Hyowon Gweon, Elizabeth S. Spelke

**Affiliations:** Department of Psychology, Harvard University, Cambridge, MA, USA; The Center for Brains, Minds, and Machines, Cambridge, MA, USA; Department of Psychological and Brain Sciences, Johns Hopkins University, Baltimore, MD, USA; Department of Psychology, Stanford University, Stanford, CA, USA

**Keywords:** cognitive development, action understanding, helping, toddlerhood, social cognition

## Abstract

Capacities to understand and evaluate others’ actions are fundamental to human social life. Infants and toddlers are sensitive to the costs of others’ actions, infer others’ values from the costs of the actions they take, and prefer those who help others to those who hinder them, but it is largely unknown whether and how cost considerations inform early understanding of third-party prosocial actions. In three experiments (*N* = 94), we asked whether 16-month-old toddlers value agents who selectively help those who need it most. Presented with two agents who attempted two tasks, toddlers preferentially looked to and touched someone who helped the agent in greater need, both when one agent’s task required more effort and when the tasks were the same but one agent was weaker. These results provide evidence that toddlers engage in need-based evaluations of helping, applying their understanding of action utilities to their social evaluations.

## INTRODUCTION

Human social life centers around coordinating one’s actions with those of others (Sebanz et al., 2006; Tomasello & Carpenter, 2007). To coordinate such actions effectively, people need to appreciate both the challenges that others face and their capacities for meeting those challenges. Adults can understand, for example, that a person who tries to move five boxes of equal size and weight faces a harder task than one who tries to move just one of the boxes, and that a person in their prime will find both tasks easier than will a young child. Based on this understanding, adults can infer who is more in need of help and direct their actions accordingly.

Even though such inferences come naturally to most adults, they involve a rather sophisticated understanding of how one agent’s social actions impact the cost faced by another agent who seeks to achieve a goal. There are at least two ways in which one might represent the costs of achieving a goal. First, some goals are objectively harder than others for a given agent (agent-general costs) due to differences in the work that must be undertaken to accomplish them. Second, some agents must work harder than others to achieve a given goal (agent-specific costs) due to differences in their abilities (e.g., strength, competence, skill, or maturity). The present experiments investigate whether toddlers evaluate helpers by considering the needs of those whom they help, defined both by agent-general and agent-specific costs.

### Representations of Action Cost and Evaluations of Social Actions

A growing body of literature demonstrates that people expect others to maximize their utilities, minimizing the costs of their actions while maximizing the rewards over their goals (i.e., Naïve Utility Calculus (Jara-Ettinger et al., 2016, 2020)) and that these expectations are present even in the first year (Gergely & Csibra, 2003; Gergely et al., 1995; Liu & Spelke, 2017). For example, infants as young as 3 months expect an agent to take a direct path to its goal and look longer—a sign of a state of surprise or a prediction failure—when instead the agent takes a longer path (Liu et al., 2019; Skerry et al., 2013); 10-month-old infants infer the value of an agent’s goal from the cost of attaining it (Liu et al., 2017) (e.g., the slopes of hills that an agent is willing to climb); and 15-month-old toddlers expect others to preferentially approach individuals who take more direct paths to their goals, and show those preferences themselves (Colomer et al., 2020). Taken together, these studies provide evidence for an early-emerging ability to use representations of physical cost to interpret other individuals’ past actions and to predict their future actions.

This body of research, however, has focused mostly on inferences about a single agent whose actions only have consequences for its own utility. Relatively little is known about whether and how such representations inform an understanding of social actions and social evaluation in young children. There is evidence that children as young as two years of age consider agents’ relative competence when they evaluate individuals who refuse to help (Jara-Ettinger et al., 2015). Here we ask whether toddlers consider the features of the beneficiaries of acts of helping when evaluating which actor is more helpful—specifically, how much help a potential beneficiary needs, defined either over the objective, agent-general cost of their attempted action or over their agent-specific ability to complete that action.

We build upon a body of experiments that have found that infants and toddlers show an early capacity for social evaluation: They preferentially look to and reach for agents who help others over agents who hinder others in the pursuit of their goals (Choi & Luo, 2023; Hamlin & Wynn, 2011; Hamlin et al., 2007, 2010; Hamlin, Ullman, et al., 2013; Margoni & Surian, 2018; Schlingloff et al., 2020; Woo et al., 2022). Although there have been failures to replicate these findings (Salvadori et al., 2015; Schlingloff et al., 2020), a meta-analysis of experiments probing early social evaluations has found that infants and toddlers demonstrate a significant preference for helpers over hinderers (Margoni & Surian, 2018). Following this meta-analysis, at least 13 additional studies have provided evidence that infants and toddlers engage in social evaluation (Woo et al., 2022). We believe that the present experiments will shed further light as to whether and under what circumstances toddlers engage in social evaluation.

Studies in this literature have provided evidence that infants and toddlers are sensitive to the intentions underlying social actions (Geraci et al., 2022; Geraci & Surian, 2023; Hamlin, Mahajan, et al., 2013; Hamlin, Ullman, et al., 2013; Strid & Meristo, 2020; Woo et al., 2017; Woo & Spelke, 2022, 2023b) and to the contexts in which social actions takes place (Hamlin, 2014; Hamlin et al., 2011; Hamlin, Mahajan, et al., 2013) (e.g., agents’ group membership and history of social behavior). To determine whether these young children are also sensitive to who needs more help, we asked whether toddlers would engage in need-based evaluations and prefer agents who choose to help someone in greater need over agents who choose to help someone in less need of help.

Within the framework of the Naïve Utility Calculus, prosocial actions like helping and teaching can be formalized as one agent adopting or taking on another agent’s utility function (Bridgers et al., 2020; Powell, 2022): Specifically, one agent (a “helper” or a “teacher”) benefits another agent (a “beneficiary”) by engaging in an action that increases the beneficiary’s rewards and reduces the beneficiary’s costs. Yet, this formalization does not readily explain how we might evaluate the relative helpfulness of actions that vary in their consequences for those involved. Studies have shed light on how older preschoolers and children evaluate the relative helpfulness of actions. These studies have found that notions of agent-general cost enter into older preschoolers’ and children’s understanding of prosocial actions (Bridgers et al., 2020; Hepach & Tomasello, 2020; Paulus, 2020; Radovanovic et al., 2023; Zhao & Kushnir, 2023). For example, 3- to 5-year-old children understand that an agent should help someone who faces a harder task than someone who faces an easier task, even though the agent should choose to do an easier task for herself when rewards are matched (Bennett-Pierre et al., 2018). If toddlers have a similar understanding of how agents help others, then toddlers’ evaluations of helping should also be sensitive to the utilities of those who are helped: They should evaluate acts of helping in relation to the needs of those who stand to benefit from the helpful action, defined over both agent-general and agent-specific costs.

### Research Overview

Here, we test whether 16-month-old toddlers evaluate helpful actions by considering the needs of their beneficiaries. We target 16-month-old toddlers, because children at this age (if not earlier) are both capable of understanding others’ actions in terms of their utilities (Gergely et al., 1995; Liu et al., 2019; Skerry et al., 2013) and of forming social preferences based on others’ social actions and intentions (Geraci et al., 2022; Geraci & Surian, 2023; Hamlin et al., 2007; Hamlin, Ullman, et al., 2013; Powell & Spelke, 2018; Thomas & Sarnecka, 2019; Woo et al., 2017; Woo & Spelke, 2022, 2023b). Our experimental design integrates methods from past experiments that serve to assess infants’ and toddlers’ understanding of objective action cost (Liu et al., 2017) (by manipulating the slope of hills), object weight (Kotovsky & Baillargeon, 1994, 1998) (by manipulating the sizes of objects), and helping (Hamlin et al., 2007, 2010) (by depicting agents who are helped or hindered in their efforts to climb hills). Specifically, we ask whether toddlers prefer helpers who choose to help someone with a task that is difficult for them over someone with a task that is easy for them to achieve on their own.

**Figure 1.**
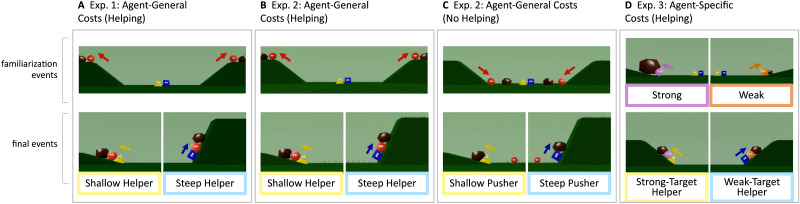
Still frames from the events presented in Experiments 1 (A), 2 (B, C), and 3 (D). Arrows are color-coded to indicate the active agent. In Experiment 1’s familiarization (A), two agents (red circles) successfully pushed boulders up hills of medium slopes while two helper agents (a yellow triangle and a blue square) watched. In Experiment 1’s final events, the circular agents simultaneously tried to push boulders up two different hills, one steeper and the other shallower than the hills in familiarization. Then, in alternating events, one helper (here, the triangle) aided the agent at the shallow hill, and the other (here, the square) aided the agent at the steep hill (see Supplemental Material for counterbalancing). Events in Experiment 2’s Helping Condition (B) were similar to those from Experiment 1 (see main text and Methods). The events in Experiment 2’s No Helping Condition (C) removed the impression of helping by constraining the circular agents to move only downward during familiarization. In the No Helping Condition’s final events, the circular agents moved down hills that were equal in slope to the shallow and steep hills of Experiment 1 and Experiment 2’s Helping Condition, and then they moved away from the hills. In alternating events, one pusher (here, the triangle) pushed the boulder at the shallow hill, and the other (here, the square) pushed the boulder at the steep hill. In Experiment 3’s familiarization (D), one circular agent (purple) demonstrated it was stronger than the other circular agent (orange) by pushing a larger boulder up a shallow hill; both agents failed to push their respective boulders up a steeper hill (not shown). Then the weak and strong agents simultaneously tried to push boulders of an intermediate size up two hills, and in alternating events, one helper aided the strong agent, and the other aided the weak agent.

In Experiments 1 through 3, two agents sought to push a boulder up a hill (Figure 1). In Experiments 1 and 2, we manipulated which of these agents (potential beneficiaries of helping) needed more help based on agent-general cost: One hill was steeper than the other. In Experiment 3, we manipulated who needed more help based on agent-specific cost: One agent demonstrated, by its past actions, that it was stronger than the other. If toddlers consider need, defined over agent-general and agent-specific costs, in their evaluations of helpers, then they should prefer the helper who took on the cost of the agent facing a steeper hill in Experiments 1 and 2, and the helper who took on the cost of the weaker agent in Experiment 3.

## EXPERIMENT 1: EVIDENCE OF EVALUATIONS BASED ON AGENT-GENERAL COST

We began by testing whether 16-month-old toddlers prefer an agent who has helped another agent climb a steep incline over an agent who has helped an agent climb a shallow incline.

### Method

Stimuli, data, and code are available on the Open Science Framework (OSF) at https://osf.io/uqa8f/.

#### Participants.

Participants were tested with their caregivers’ informed consent in all three experiments. All study protocols were approved by Harvard University’s Committee on the Use of Human Subjects. Participants received $5 USD and a certificate of participation for their participation; in-person participants also received a small prize (e.g., a stuffed animal).

In Experiment 1, twenty-two 16-month-old toddlers contributed data (mean age = 15.86 months; range = 15;14–16;18; 10 girls, 12 boys). Two of these participants began the experiment but did not produce data for the preference test due to fussiness (*n* = 1) or failure to reach for an agent (*n* = 1). Experimenters who were naïve to the events seen by participants determined these exclusions using preregistered criteria. We were able to retain their data, however, for analyses of looking time in the final events.

Participants were recruited through phone calls or emails to caregivers listed in the laboratory’s database of families who had expressed interest in participating in developmental research (e.g., by responding to mailings or signing up online). In Experiment 1, all participants came from the greater Boston area. Approximately 90% of these participants’ caregivers completed demographics questionnaires. Of these participants, 70% were White, 10% were Asian, 5% were Hispanic, and 10% were multiracial.

#### Sample Size Justification.

Experiment 1 was not formally preregistered, but its sample size was planned prior to data collection. For all our experiments, sample sizes were similar to that of prior research on 2-year-old toddlers’ evaluations of agents in relation to their competence (Jara-Ettinger et al., 2015).

#### Displays.

Each toddler viewed 4 familiarization events and 4 final events, for a total of 8 events. All events were created in Blender and depicted two red circular agents that engaged in goal-directed motion, and either one or two helper agents (a yellow triangular agent and a blue square agent) who aided them. All agents were colored shapes with eyes, and the events took place in a scene with two hills, one on each side of the screen. Two previous studies have used two-hill displays to study social cognitive development in infants (Geraci et al., 2022; Geraci & Surian, 2023).

#### Familiarization Events.

Toddlers were first familiarized to videos of two red circular agents (the potential beneficiaries) who pushed boulders up two distinct hills with equal slopes (37.5 degrees, henceforth the “medium” hill) as two animated agents of different shapes and colors (the helpers; one square and one triangle) observed (Figure 1A). In each familiarization event, the two red agents began in the center of the screen, at the base of two medium 37.5-degree hills, that rose in opposite directions. One after the other, both red agents tried to climb the hills that they were stationed near, as the square and the triangle observed. A boulder rolled down the slope of the hill, pushing the circular agent down to the base of the hill. The agent tried to climb again and eventually pushed the boulder to get to the top of the hill. Following these events on one hill, the same events occurred at the remaining hill with the other circular agent, as the square and the triangle observed. As soon as the second agent pushed the boulder to the top of the hill, we proceeded to the next event.

#### Final Events.

In each final event, the circular agents faced two new hills: One hill (60 degrees) was steeper than the one in familiarization, and the other hill (15 degrees) was shallower than the one in familiarization. In each event, one helper (either the triangle or square) was present to watch the two circular agents simultaneously try to climb these new hills. As before, boulders (of equal size) rolled down on the circular agents, and they tried to resist them as the helper looked on. The agent facing the shallow hill had an objectively easier task that it should be able to perform without aid, for in familiarization, it had independently climbed a hill that was steeper than the shallow hill; thus, it was not as much in need of help as the agent facing the steep hill, whose task was objectively harder than the one it had performed earlier.

In alternating events, each of the helpers consistently chose to aid one of the other agents, pushing it up slightly. One helper always helped the agent at the steeper hill, whereas the other helper always helped the agent at the shallower hill. We will call the helpers the Steep Helper and the Shallow Helper. In each final event, motion stopped when the helper started pushing the beneficiary, to equate motion across the two beneficiaries within each event. Although the helpers did not push the beneficiaries all the way up the hill, past research has found that by late in the first year, infants infer intentions to help from such attempts to help (Hamlin, Mahajan, et al., 2013).

#### Procedure.

Toddlers sat on their caregiver’s lap in the lab before a LCD projector screen 102 cm in height and 132 cm in width.

After toddlers watched all the events, we assessed toddlers’ preference between the Steep and Shallow Helpers via their preferential reaching. An experimenter (who was blind to the events) presented toddlers with physical, paper facsimiles of the Steep and Shallow Helpers in a reaching choice test. Specifically, the experimenter held up both helpers on a board, approximately 30 cm apart and out of reaching distance, and first said, “Look!” After toddlers had looked at both helpers, the experimenter said “Hi” to recenter toddlers’ gaze, and moved the board forward, and asked, “Who do you like?” Choice was determined by this experimenter as the first helper toddlers touched by means of a visually guided reach (i.e., the first helper that toddlers looked at and then reached for). A second experimenter who was unaware of the events that toddlers had seen also judged which helper toddlers reached for. There was 100% agreement between the two sets of judgments. For all experiments, see Supplemental Material for counterbalancing.

#### Coding of Looking in the Final Events.

We measured toddlers’ looking times to the final events for exploratory analyses to probe toddlers’ interest or expectations of helping (i.e., violation-of-expectation) (see Supplemental Material). In past research, infants and toddlers typically have not looked longer to events involving helping vs. hindering (Hamlin et al., 2007, 2010; Woo et al., 2017), but infants and toddlers have looked longer to events involving unfair action vs. fair actions (Buyukozer Dawkins et al., 2019; Schmidt & Sommerville, 2011; Sloane et al., 2012), suggesting that they expect agents to behave fairly.

As soon as the helper chose to push one agent and boulder up one of the two hills, all motion was paused, and an experimenter (blind to the events) began coding the final events, using the coding program XHAB (Pinto, 1996). The coder coded toddlers’ looking until the end of each final event, when 30 cumulative seconds had passed, or toddlers had looked away for 2 consecutive seconds. To test reliability, we randomly selected 25% of toddlers and had their final events coded by an additional coder who was unaware of experimental condition and of the events that toddlers had seen. The intraclass correlation between the coders’ times was 0.97.

### Results

**Figure 2.**
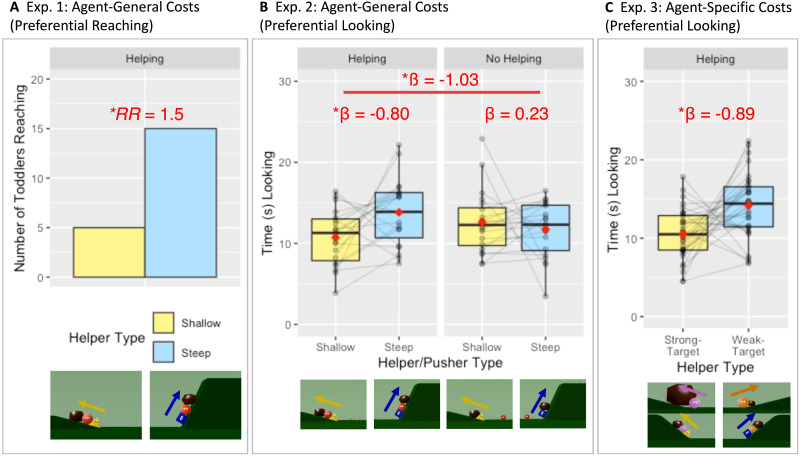
Results in Experiment 1 assessing preferential reaching (A), and in Experiments 2 and 3 assessing preferential looking (B, C). The images below the graphs are representative of the displays during the final events (see Supplemental Material for counterbalancing). Panel A depicts the number of toddlers choosing the helpers on the shallow and steep slopes in Experiment 1. RR indicates relative risk. Panels B and C respectively indicate the amount of time that toddlers looked to the agents (helpers or pushers) in Experiments 2 and 3. In Panels B and C, red diamonds indicate means, pairs of connected dots indicate data from a single toddler, horizontal lines within boxes indicate medians, boxes indicate interquartile ranges, and whiskers indicate 1.5 times the interquartile range. The beta coefficients (*β*) indicate standardized effect sizes (following corrections for multiple comparisons in Experiment 2). Asterisks indicate significant differences (*p < .05; 2-tailed).

Toddlers reached more to the Steep Helper over the Shallow Helper (15/20 toddlers chose the Steep Helper, binomial *p* = .041, relative risk = 1.5; Figure 2A). This choice likely did not reflect greater interest or surprise at events involving the Steep Helper, because exploratory analyses found that toddlers did not look differently at the final events depicting helping at the steep hill (mean_steep-final-event_ = 7.83 s, *SD* = 6.58 s) and helping at the shallow hill (mean_shallow-final-event_ = 8.04 s, *SD* = 6.84 s; *β* = 0.09, 95% CI of *β* [−0.17, 0.36], *b* = 0.10, *t*(44) = 0.69, *p* = .493; see Supplemental Material for full details).

### Discussion

Experiment 1 suggests that 16-month-toddlers prefer an agent who helps someone who faces a harder task and therefore is in greater need of help and would benefit more greatly by being helped. However, an alternative interpretation of these findings is that toddlers considered only each agent’s choice to expend physical effort, without integrating this cost with benefit for the agent in need. Given that 2-year-old children’s social preference can be influenced by competence (Jara-Ettinger et al., 2015), children in our study might have preferred agents who chose to perform the more difficult task, because the choice to perform a harder task suggests greater strength or competence. We conducted Experiment 2 to replicate Experiment 1 and test this alternative explanation.

## EXPERIMENT 2: FURTHER EVIDENCE OF EVALUATIONS BASED ON AGENT-GENERAL COST

Experiment 2 conceptually replicated Experiment 1 and further tested whether the toddlers’ performance in Experiment 1 reflected their evaluation of the agents as helpers or as competent actors. Due to the COVID-19 pandemic, we conducted Experiments 2 and 3 using online methods via a video conference software (Zoom). Because we could not elicit reaching behavior to physical objects over video calls, we instead probed toddlers’ evaluations by measuring how long toddlers chose to look at the two helpers using a preferential looking test, following prior research that used this measure to assess infants’ social evaluation in traditional laboratory settings (Hamlin & Wynn, 2011; Hamlin et al., 2010) and more recently in online experiments with infants and toddlers (Woo & Spelke, 2023a, 2023b).

We randomly assigned 16-month-old toddlers to two conditions. In the Helping Condition (*n* = 20; Figure 1B), methods were highly similar to those of Experiment 1 (Figure 1B; see Method and Supplemental Material). In the No Helping Condition (*n* = 20; Figure 1C), we removed the impression that agents were helping in final events in two ways. First, the characters who needed help in Experiment 1 were present during familiarization but never demonstrated the goal of climbing hills; instead, they each descended a hill. Second, the agents now only pushed the boulders, not another agent, in the final events of this condition. Thus, we assessed whether toddlers preferred the agent who chose to engage in the harder task, without helping someone else. Because no helping was involved in the No Helping Condition, we refer to the two agents as “pushers” rather than as “helpers”. If the toddlers in Experiment 1 had evaluated the helpers based on competence, then the toddlers in Experiment 2 should prefer the Steep Actor in both the Helping and No Helping Conditions. If the toddlers in Experiment 1 had engaged in need-based evaluations, then the toddlers in Experiment 2 should only prefer the Steep Actor in the Helping Condition.

### Method

Experiments 2 was preregistered on the OSF at https://osf.io/uqa8f/.

#### Participants.

In Experiment 2, forty 16-month-old toddlers contributed data (mean age = 16.24 months; range = 15;13–16;25; 23 girls, 17 boys). One of these participants began the experiment but did not produce data for the preference test due to fussiness (*n* = 1). We were able to retain the data, however, for analyses of looking time in the final events. An additional toddler began the experiment but was not included in the final sample due to distractions in the home (*n* = 1).

In Experiment 2, online participation allowed us to test infants outside of [North American city hidden]. In Experiment 2, participants came from the greater Boston area (*n* = 36), or from online signups (*n* = 4) over ChildrenHelpingScience.com. Approximately 43% of participants completed demographics questionnaires. Approximately 52% of these participants were White, 18% were Asian, 18% were multiracial, 6% were Black, and 6% were Hispanic.

#### Sample Size Justification.

We had preregistered a sample size of 32 toddlers for Experiment 2 (i.e., 16 per condition), based on power analyses using pilot data (see preregistration for full details). However, more families responded than we had anticipated, resulting in a sample of 40. Data analyses were only performed after all data were collected.

#### Displays.

Each toddler viewed 1 familiarization event and 6 final events, for a total of 7 events. The Helping Condition was nearly identical to Experiment 1, except that the hills now had blades of grass to better convey depth (which were useful for the No Helping Condition), and the number of final events differed (see Figure 1B). The No Helping Condition was well matched to the Helping Condition in terms of the number of agents and objects, the costs of actions, and task difficulty, but we removed the impression of helping in two ways (see Figure 1C). First, we changed the movements of the circular agents such that each moved down rather than up a hill, a boulder fell down the sky away from the hills, and the agent tapped the boulder. Critically, because the circular agent never tried to climb up the hill with the boulder, and never even moved in the direction of the hill with the boulder, it should not appear to have the goal of pushing the boulder up the hill. Second, during the final events, the circular agent moved down the hills and moved backwards, as though disengaging from the boulders and the hills. The two “pushers” (the triangle and square) chose to either push the boulder up either the steep or the shallow hill.

In both conditions, the videos looped as soon as the center agent chose one of the hills and began pushing a circular agent and a boulder (in the Helping Condition) or just the boulder (in the No Helping Condition). When adapting this study for online testing, we chose to loop videos to better engage toddlers over video calls. Although toddlers never saw agents fully climb hills in the No Helping Condition’s final events, toddlers should nevertheless be capable of inferring that the agents intended to act on one hill over another, given past research suggestive that infants infer goals from such selective actions (Woodward, 1998), that infants infer the goals of incomplete actions (Hamlin et al., 2008), and that infants infer the goal of an agent who tries but fails to climb a hill (Hamlin et al., 2007, 2010).

#### Procedure.

Data collection occurred in toddlers’ homes, with toddlers observing the events displayed via screen-sharing over Zoom on a caregiver’s personal electronic device (e.g., laptops). Toddlers either sat on a caregiver’s lap or on a highchair, with the caregiver not in the toddler’s view. Caregivers received instructions to set-up the experiment and optimize data collection: making displays full screen, ensuring toddlers’ eyes were visible on camera, hiding toddlers’ webcam view of themselves, and reducing adult influence.

After the toddlers watched all the events, we assessed toddlers’ preference between the Steep and Shallow Actors (i.e., helpers in the Helping Condition, pushers in the No Helping Condition) by measuring their preferential looking. We first used attention grabbers to determine how the toddlers looked as they were looking left and right, and then used a final attention grabber to recenter toddlers’ gaze. Next, we presented the toddlers with a 30-second video, in which the Steep and Shallow Actors appeared on opposite sides of the screen and moved to an experimenter’s prerecorded voice saying “Hi! Look! Who do you like?” 3 times, once every 10 seconds. An experimenter, who was unaware of the events and condition, coded the video of the toddlers from this portion of the study to determine how much time the toddlers had spent looking at each actor.

Preferential looking in the choice test differed from the looking after the final events in several ways. First, whereas the choice test lasted 30 s regardless of how long a toddler attended to the video, the looking time measures in the final events ended when a toddler had looked away for 2 consecutive s. Second, whereas the choice test tracked which side of the displays the toddler looked to, the looking time measures in the final events involved any part of the scene during the final events. Third, whereas the choice test occurred after the toddlers had seen all the events, the coding of looking in the final events occurred during those events. Finally, whereas the choice test occurred as the agents faced the toddler, accompanied by social language (a voice asking, “Who do you like?”), the looking time measures in the final events occurred when the helpers acted, with no accompanying social language. Thus, the choice test was more socially directed at the toddler than were the final events; we take the choice test as a measure of social engagement with the different agents, and the final event looking times as a measure of interest in or processing of each of the full final events. These interpretations are consistent with past research in which infants and toddlers looked longer at prosocial agents, but attended equally to events in which agents engaged in prosocial and antisocial actions (Hamlin et al., 2010; Hamlin & Wynn, 2011; Woo & Spelke, 2023a, 2023b).

#### Coding of Looking in the Final Events.

In both conditions’ final events, as soon as the center agent chose one of the hills and began pushing a circular agent and a boulder (in the Helping Condition) or just the boulder (in the No Helping Condition), the videos looped, and an experimenter (blind to events) began coding the final events using the coding program jHab (Casstevens, 2007). The coder coded the toddlers’ looking until the end of each final event, when 45 cumulative seconds had passed, or the toddlers had looked away for 2 consecutive seconds.

#### Reliability.

To test reliability, we randomly selected 25% of the toddlers and had their final events and choice tests coded by an additional coder who was unaware of experimental condition and of the events that the toddlers had seen. The intraclass correlation between the coders’ times was 0.99 for the final events. The intraclass correlations were 0.90 and 0.95 for looking left and looking right, respectively, in the choice test.

### Results

In preregistered analyses, we ran a mixed-effects model to determine whether raw preferential looking times differed during the choice test for the Steep and Shallow Actors within each condition. We found that the toddlers’ looking between the Steep and Shallow Actors differed across the Helping and No Helping Conditions (*β* = −1.03, 95% CI of *β* [−1.88, −0.19], *b* = −3.98, *t*(78) = 2.40, *p* = .018). Post-hoc pairwise comparison tests, correcting for multiple comparisons using Holm’s method, revealed that the toddlers looked longer to the Steep Helper (mean_steep-choice_ = 13.82 s, *SD* = 4.17 s) over the Shallow Helper (mean_shallow-choice_ = 10.73 s, *SD* = 3.49 s) in the Helping Condition (*β* = −0.80, *b* = −3.09, *t*(41) = −2.53, *p* = .015), but did not show a preference between the Steep Pusher (mean_steep-choice_ = 11.72 s, *SD* = 3.40 s) and the Shallow Pusher (mean_shallow-choice_ = 12.61 s, *SD* = 3.92 s) in the No Helping Condition (*β* = 0.23, *b* = 0.88, *t*(41) = 0.747, *p* = .459, Figure 2B). Consistent with these results, the toddlers also spent a greater proportion of time looking at the Steep Actor in the Helping Condition, but not in the No Helping Condition (see Supplemental Material for full details).

Did these looking preferences reflect the toddlers’ greater interest in the agent who acted on the steeper hill, because they have expectations about which of these actions the agents would choose to perform? To answer this question, we first conducted a preregistered, exploratory analysis on the toddlers’ looking times at the final events, testing whether the toddlers looked differently when agents acted on the steep or shallow hill. In the model, we included event type (Shallow/Steep), condition (Helping/No Helping), and their interaction as fixed effects. As in Experiment 1, we found that the toddlers did not look differently to the Steep (mean_steep-final-event_ = 22.96 s, *SD* = 17.22 s) and the Shallow (mean_shallow-final-event_ = 23.70 s, *SD* = 17.30 s) events in the Helping Condition. Toddlers also did not look differently to the Steep (mean_steep-final-event_ = 26.54 s, *SD* = 16.79 s) and the Shallow (mean_shallow-final-event_ = 26.86 s, *SD* = 17.37 s) events in the No Helping Condition. Neither event type nor condition significantly predicted looking times in the final events (*p*s > .73), and their interaction was not significant (*p* = .891) (see Supplemental Material for full details and further analyses demonstrating that looking in the final events did not predict looking in the choice test). The toddlers did not expect either helpers or pushers to choose the more costly action.

### Discussion

In Experiment 2, toddlers again preferred an agent who helped someone facing a hard task over an agent who helped someone facing an easy task. In contrast, when the same agents engaged in a task that did not involve helping, toddlers did not prefer an agent who engaged in a hard task over an agent who engaged in an easy task. These results conceptually replicate the findings from Experiment 1 using a different testing method (online) and dependent variable (preferential looking). Importantly, the lack of effect in the No Helping Condition provides evidence against the possibility that the toddlers preferred to reach for and look at the Steep Helper because its action was more expected, or because it demonstrated greater strength, competence, or motivation for hard work. Toddlers’ preference for the Steep Actor was manifest only in a social context involving agents who could be helped.

In Experiments 1 and 2, we sought to establish the agents’ needs for help by manipulating agent-general costs imposed by the external environment: the slope of the hills that the agents attempted to climb. As adults, however, we evaluate helpers based not only on such agent-general costs, but on factors that are internal to the agents themselves (agent-specific cost). In Experiment 3, we tested whether toddlers’ social evaluations are sensitive to such agent-specific costs by presenting agents who faced tasks of equal objective difficulty, but whose need for help varied due to their difference in competence. If toddlers’ evaluations in Experiments 1 and 2 reflect an understanding of which agent was in greater need of help, then toddlers may also prefer someone who helps the less competent agent in Experiment 3.

## EXPERIMENT 3: EVALUATIONS BASED ON AGENT-SPECIFIC COST

Experiment 3 investigated whether 16-month-old toddlers prefer an agent who has helped a weak individual over an agent who has helped a strong individual. The displays and actions were similar to those in Experiments 1–2. The main difference was that the toddlers were first presented with videos of two agents pushing boulders of different sizes to establish that one was stronger than the other (Figure 1D). In these familiarization videos, both agents successfully pushed their respective boulders up the shallow hill (15 degrees) and failed to push the same boulders up the steep hill (60 degrees), but one agent (the Strong Beneficiary) always pushed a large boulder (1.15 units of Blender space in diameter) whereas the other (the Weak Beneficiary) always pushed a small boulder (0.3 units in diameter). Despite the large boulder ostensibly being heavier, the agent acting on that boulder was able to push with the same ease as the agent pushing the small boulder. Thus, we provided information that one agent was stronger than the other.

In the final events that followed this familiarization, both the weak and strong agents simultaneously tried to push medium-sized boulders (0.6 units in diameter, intermediate in size to the boulders from familiarization) up the medium hills (37.5 degrees). The sizes of the boulders in these events were proportional to those used in to experiments to test toddlers’ understanding of weight in relation to size (Kotovsky & Baillargeon, 1994, 1998). Thus, even though both agents faced identical tasks, insofar as toddlers understood the familiarization events, they would expect that the task is harder for the weak agent. In alternating final events, one agent pushed the Weak Beneficiary, whereas the other agent pushed the Strong Beneficiary. Following all the events, we assessed toddlers’ evaluations of the two helpers in a preferential looking choice test, as in Experiment 2.

### Method

Experiments 3 was preregistered on the OSF at https://osf.io/uqa8f.

#### Participants.

In Experiment 3, thirty-two 16-month-old toddlers contributed data (mean age = 16.10 months; range = 15;12–16;26; 16 girls, 16 boys). Two of these participants began the experiment but were not included in the final sample due to distractions in the home (*n* = 1) and connection issues with the video conferencing (*n* = 1).

In Experiment 3, participants came from online signups (*n* = 4), or over ChildrenHelpingScience.com. Approximately 41% of participants completed demographics questionnaires. Approximately 38% of these participants were White, 31% were Asian, and 31% were multiracial.

#### Sample Size Justification.

We had preregistered a sample size of 32 toddlers for Experiment 3, based on power analyses using pilot data (see preregistration for full details).

#### Displays.

Each toddler viewed 6 familiarization events and 6 final events, for a total of 12 events. The goal of familiarization was to convey that one circular agent was stronger than the other. In familiarization, both agents tried to push boulders up hills. Whereas the stronger agent acted on a large boulder (1.15 units of Blender space in diameter), the weaker agent instead acted on a small boulder (0.3 units in diameter).

#### Familiarization Events.

In each familiarization event, one circular agent (either pink or orange, in alternating events) tried to climb hills on one side of the screen, and two other agents (a square and a triangle) were present at the center of the screen to watch the circular agent act on the hills (Figure 1D). The first hill in each event was 15 degrees, and the circular agent tried to climb it. A boulder (varying in diameter, as above) rolled down the slope of the hill, pushing the agent down. The agent stopped the boulder and pushed it to get to the top of the hill. Immediately after reaching to the top of the hill, each event depicted a second, steeper hill (60 degrees), and the circular agent again tried to climb it but was pushed down by a boulder. With this steeper hill, the agent was not able to overcome the boulder. Then, the videos looped.

#### Final Events.

In each final event, there was one hill (37.5 degrees; of intermediate angle between the shallow and steep hills) on each side of the screen, and for the first time, both the weaker and the stronger circular agents simultaneously tried to climb the hills. As before, boulders (0.6 units in diameter) rolled down on the agents, who tried to reverse their motion, as a single agent (either the square or the triangular helper) observed. In alternating events, one helper always aided the weak agent, whereas the other helper always aided the strong agent. We refer to the helpers as the Weak-Target and Strong-Target Helpers, respectively. As soon as a helper began pushing a beneficiary in the final events, the videos looped.

#### Procedure.

The procedure was like that of Experiment 2, except that the preferential looking test involved the Weak-Target and Strong-Target Helpers.

#### Coding of Looking in the Final Events.

As soon as a helper began pushing a beneficiary in the first loop of a final event, an experimenter (blind to the events) began coding using jHab (Casstevens, 2007). The coder coded toddlers’ looking until the end of each final event, when 30 cumulative seconds had passed, or toddlers had looked away for 2 consecutive seconds. (We chose to have a shorter maximum coding time for final events in Experiment 3, to reduce fussiness and inattentiveness given that there were more familiarization trials than in Experiment 2.)

#### Reliability.

We randomly selected 25% of the toddlers and had their final events and choice tests coded by an additional coder who was unaware of the events that toddlers had seen. The intraclass correlation between the coders’ times were 0.99 for the final events. The intraclass correlations were 0.93 and 0.96 for looking left and looking right, respectively, in the choice test.

### Results

Using a mixed-effects model to determine whether raw preferential looking times differed during the choice test for the Weak-Target and Strong-Target Helpers, we found that toddlers looked longer to the Weak-Target Helper (mean_weak-target-choice_ = 14.20 s, *SD* = 4.10 s) than to the Strong-Target Helper (mean_strong-target-choice_ = 10.53 s, *SD* = 3.27 s) (*β* = 0.89, 95% CI of *β* [0.46, 1.32], *b* = 3.66, *t*(64) = 4.01, *p* < .001) (Figure 2C). Again, in line with our earlier exploratory findings, toddlers did not expect helpers to help the Weak Beneficiary rather than the Strong Beneficiary: They looked no longer when the Weak Beneficiary received help (mean_strong-final-event_ = 14.03 s, SD = 10.75 s) than when the Strong Beneficiary received help (mean_strong-final-event_ = 14.70 s, *SD* = 9.53 s) (*β* = 0.07, 95% CI of *β* [−0.16, 0.29], *b* = 0.67, *t* = 0.57, *p* = .569). See Supplemental Material for full details.

### Discussion

Toddlers in Experiment 3 preferred an agent who helped a weaker individual over an agent who helped a stronger individual. These findings conceptually replicate those of Experiments 1 and 2, in a situation in which we established need not by manipulating the slopes of the hills that beneficiaries of helping sought to climb, but by manipulating each beneficiary’s ability to complete the same task on its own. Together with Experiment 1 and 2, these findings provide evidence that toddlers’ social evaluations are modulated both by others’ needs and by the agent-general and agent-specific costs of the beneficiaries of helping.

## GENERAL DISCUSSION

Across three experiments, 16-month-old toddlers evaluated agents who helped others based on how they responded to others’ needs. In Experiment 1, toddlers preferred an agent who helped someone trying to climb a steep hill over an agent who helped someone trying to climb a shallow hill. Experiment 2 replicated this finding and further established that such a preference is specific to prosocial interactions; when agents simply engaged in a harder or easier task, toddlers did not show a clear preference, ruling out the possibility that toddlers simply prefer an agent who chooses to perform a harder task. Finally, in Experiment 3, toddlers preferred an agent who helped the weaker of two potential beneficiaries over an agent who helped the stronger one, when the two beneficiaries engaged in the same task. Taken together, these results provide evidence that toddlers’ evaluations of helpers take account of both the agent-general and agent-specific costs faced by their beneficiaries. They build on and extend the growing body of research on the early development of reasoning about others’ costs and rewards in social contexts (Bennett-Pierre et al., 2018; Bridgers et al., 2020; Colomer et al., 2020; Jara-Ettinger et al., 2015; Liu et al., 2017; Zhao & Kushnir, 2023), providing evidence that representations of cost enter into early understanding of social actions.

The present results also build on and extend the growing body of research on the early development of social evaluation, providing evidence that toddlers engage in need-based evaluations of helping. Past research has posited that primitive abilities to make sense of prosocial and antisocial behaviors emerge in infancy (Hamlin, Ullman, et al., 2013; Ting et al., 2020; Woo et al., 2022); the present experiments shed light on the depth of these abilities in toddlerhood. We look forward to research that further applies the Naïve Utility Calculus framework to children’s understanding of social interactions. We have focused on a prosocial action (helping), and it is unknown whether infants or toddlers also make sense of antisocial actions (e.g., hindering) in terms of action cost.

These findings are noteworthy for at least two reasons. First, whereas prior research has provided evidence that infants and toddlers reason about agent-general costs as determined by the features of the external environment (Gergely et al., 1995; Liu et al., 2019; Liu & Spelke, 2017; Skerry et al., 2013), the results from Experiment 3 suggest that toddlers can reason about agent-specific costs that reflect internal properties of agents such as ability, complementing research in older children (Jara-Ettinger et al., 2015). In addition to attending to information that was immediately available about agent-general cost in Experiments 1 and 2 (the slope of hills), toddlers drew on their prior observations of agents’ abilities (to push boulders in familiarization) to evaluate acts of helping in Experiment 3. Moreover, toddlers’ looking preferences in Experiment 2’s Helping Condition and in Experiment 3 were roughly equal in size regardless of whether we manipulated agent-specific or agent-general costs, suggesting that children are equally sensitive to both dimensions of cost at 16 months. The emergence, generality, and limitations of toddlers’ understanding of action cost merit further investigation, however, through experiments that explore, both in social and nonsocial contexts, how infants, toddlers, and young children reason about agent-general vs. agent-specific costs in situations requiring finer tradeoffs than the situations used in the present experiments.

Second, despite an early-emerging expectation that agents act efficiently to minimize the costs of their own actions (Gergely et al., 1995; Liu et al., 2019; Liu & Spelke, 2017; Skerry et al., 2013) and an early-emerging preference for agents who act efficiently (Colomer et al., 2020), the toddlers in our study preferred an agent who chose to perform a more costly action for the benefit of someone else. This seeming discrepancy can be explained by extending recent proposals that apply the Naïve Utility Calculus framework to social interactions (Bridgers et al., 2020; Powell, 2022). Extending the idea that helping involves an agent “adopting” the utility of another agent (Powell, 2022), we speculate that toddlers understand the actions presented in the current paper by considering the utilities of both potential beneficiaries: the agent who needed help more and the agent who needed help less (i.e., both agents would benefit from being helped but one would benefit more). Under this interpretation, toddlers (i) computed the downstream utilities of both beneficiaries, given each helping action and (ii) chose the helper that maximized the joint utility across both beneficiaries. In our experiments, however, a rational observer may also judge that only one of the two potential beneficiaries needed help, for the other beneficiary had completed a task that was (likely to have been) more difficult earlier. It is possible that toddlers made this categorical distinction. Under either of these interpretations, toddlers evaluated helpful actions in accord with the relative needs of the actors to whom the actions were directed.

However, we also recognize another way of interpreting the present findings. Past research by Liu et al. (2017) has revealed that when an agent is willing to work hard to achieve one goal but not another (e.g., by climbing vs. refusing to climb a steep hill depending on the goal), 10-month-old infants expect the agent to prefer the goal that it had been willing to work hard to achieve. Based on the difference in action cost that the beneficiaries of helping faced, then, the toddlers may have inferred that one beneficiary more strongly valued climbing the hill that it approached. Under this interpretation, the toddlers’ evaluations of helping still reflect sensitivity to action cost, but in a different way: They would have formed evaluations based on the reward that a potential beneficiary of helping stood to gain by being helped.

Taken together, toddlers appear sensitive to the relative costs of different tasks (either agent-general or agent-specific), and they use either just the costs of those tasks or also the inferred rewards associated with those tasks in order to evaluate agents who help with those tasks. We have directly manipulated the costs that the different beneficiaries faced (and thus, the needs of the beneficiaries) in our stimuli; the rewards that they would gain must instead be inferred from the beneficiaries’ actions. It is unclear, however, whether the toddlers inferred the relative rewards of different actions when observing the present displays for two reasons.

First, there were aspects of our displays that would not support attributing different levels of reward to the different hills. The potential beneficiaries of helping only ever faced the decision of climbing one hill: the hill that they were stationed at. Whereas the agent in Liu and colleagues’ experiments refused to engage in a second possible task, the potential beneficiaries of helping never refused to engage in any tasks. Moreover, the beneficiaries never once looked at the other hill. Past research has revealed that infants and toddlers consider agents’ states of knowledge and ignorance when reasoning about others’ actions (Choi et al., 2018; Luo & Johnson, 2009). For example, after an actor reaches for one object over another, while both objects are visible to the actor, 6-month-old infants later expect the actor to continue reaching to the same object as before. However, if the actor reaches for one object, while facing away from another object that she has never seen, infants do not expect the actor to later reach for the same object as before when the objects are later both visible to the actor. Because the beneficiaries did not look to both hills in the present experiments, there was no evidence that the beneficiaries chose to act on one hill over the other; thus, the toddlers were unlikely to have inferred that each beneficiary valued the two hills differently.

Second, Experiment 3 provides evidence against the possibility that the toddlers’ evaluations were solely based on inferring that one hill was more rewarding than the other. In Experiment 3, the hills in the final events were equal in slope, and it was not until the beneficiaries had already chosen to attempt to climb a hill that the two differently sized boulders appeared. That is, the beneficiaries’ choice to begin climbing was not based on the difficulty of the hills themselves. Those choices therefore would not have supported inferences that one hill was more rewarding than the other. Nevertheless, the toddlers differently evaluated the two helpers. The evidence therefore suggests that toddlers were sensitive to each agent’s need for help. We look forward to future research that more directly distinguishes between the present interpretations.

In sum, we show that by 16 months, toddlers use the costs of others’ actions to make judgments of relative helpfulness. Beyond judging who is helping and hindering, toddlers judge who is more vs. less helpful, based on what tasks other individuals need help with and on how capably those individuals can complete those tasks on their own. Thus, like adults and young children, toddlers appear to appreciate that prosocial interactions fundamentally involve tradeoffs in utilities: that when agents help, they take on another individual’s goals and costs as their own, and by choosing to help one individual and not another, agents’ choices may have downstream consequences on both individuals’ utilities. The ability to interpret agents’ behaviors in terms of their physical constraints and abilities, their self-serving utilities, and their dispositions to help others by reducing the costs of others’ actions, may be a key foundation of our human social intelligence.

## ACKNOWLEDGMENTS

We thank the families who participated in these studies, the Cambridge Writing Group for feedback on an early proposal, Kiley Hamlin and Lindsey Powell for helpful discussion, Bill Pepe, Will Adams, Kexin Que, and Lauren Salmans for help with data coding, and ChildrenHelpingScience.com for support with recruitment.

## FUNDING INFORMATION

This work was supported by National Science Foundation STC award CCF-1231216 and by a Social Sciences and Humanities Research Council Doctoral Fellowship (752-2020-0474) (B. W.).

## AUTHOR CONTRIBUTIONS

All authors contributed to experimental concept and design. B. W. performed data collection and analysis, B. W. drafted the manuscript, H. G. contributed protocols to facilitate online testing, and S. L., H. G., and E. S. provided critical feedback and revisions to the manuscript.

## ETHICS APPROVAL STATEMENT

All study protocols were approved by the Harvard University Committee on the Use of Human Subjects.

## DATA AVAILABILITY STATEMENT

Experiments 2 and 3 were formally preregistered. All deidentified data are hosted on the Open Science Framework at https://osf.io/uqa8f/.

## Supplementary Material


