# Supplementary material for: Toddlers Prefer Agents Who Help Those Facing Harder Tasks
Source: Open Mind (Camb). 2024 Apr 10;8:483–99. doi: 10.1162/opmi_a_00129 (PMC11045033; doi:10.1162/opmi_a_00129)
Supplement: Supplementary file 1 [file opmi-08-483-s001.pdf]

Supplementary Information for

Toddlers Prefer Agents Who Help Those Facing Harder Tasks

Brandon M. Woo[1,2], Shari Liu[3], Hyowon Gweon[4], and Elizabeth S. Spelke[1,2]

[1]Department of Psychology, Harvard University, Cambridge, MA 02138;

[2]The Center for Brains, Minds, and Machines, Cambridge MA, 02139;

[3]Department of Psychological and Brain Sciences, Johns Hopkins University, Baltimore MD, 21205; and

[4]Department of Psychology, Stanford University, Stanford, CA 94305

Supplementary Methods

Hypotheses, methods, and analysis plans were preregistered on the Open Science Framework for Experiments 2 and 3 at https://osf.io/uqa8f/.

Counterbalancing

Experiment 1. The following variables were counterbalanced across toddlers: the shape and color of the Steep Helper (yellow triangle or blue square); the side of the Steep Helper in familiarization (left or right); the order in familiarization of the actions of the agent in greater need of help (first or second); the side of the steeper hill in the final events (left or right); the order of the Steep Helper in final events (first or second); and the side of the Steep Helper in the choice test (left or right).

Experiment 2. The following were counterbalanced across toddlers: the color and shape of the Steep Actor (yellow triangle or blue square), the side of the Steep Actor (left/right), and the order of actions at the steeper hill in final events (hard action first or second). For a single toddler, the Steep and Shallow Actors always stayed on the same side of the screen for the whole experiment. Because we counterbalanced the identity of the Steep Actor (who acts at the steeper hill) and the side of the steeper hill, half of the toddlers saw the triangle on the left of videos, and the other half saw it on the right.

Experiment 3.

The following were counterbalanced across toddlers: the color of the weak agent (orange or purple), the color and shape of the Weak-Target Helper (yellow triangle or blue square), the side of the Weak-Target Helper (left or right), and the order in which the Weak-Target Helper acted in final events (first or second). For a single toddler, the Weak-Target and Strong-Target Helpers always stayed on the same side of the screen for the whole experiment.

Supplementary Results

Experiment 1

To examine whether toddlers expected helpers to help at the steep or the gentle hill in the final events, we ran a mixed-effects model using the lme4 (Bates et al., 2015) and lmerTest packages (Kuznetsova et al., 2017). In the model, looking time was the dependent variable, event type (Shallow/Steep) was a fixed effect, and there was a random intercept for participant ID with final event pair (first/second) as a random slope. (Because a lognormal distribution fit these data better than a normal distribution did, we log transformed the looking time data from final events before including them in this model.) As reported in the main text, we found that toddlers did not look differently to Steep ($mean_{steep\text{-}final\text{-}event}$ = 7.83 s, SD = 6.58 s) and Shallow ($mean_{shallow\text{-}final\text{-}event}$ = 8.04 s, SD = 6.84 s) events (β = 0.09, 95% CI of β [-0.17, 0.36], b = 0.10, $t(44)$ = 0.69, p = .493).

Next, to determine whether there were alternative, low-level reasons for toddlers' reaching, we ran exploratory binomial tests. There were no significant preferences based on the side of presentation, order of presentation, or shape of helpers (ps < .50).

Experiment 2

The analyses reported in the main text on the choice test were based on analyses of raw preferential looking time. In additional preregistered analyses, we examined what proportion of time toddlers looked at the Steep Actor when they looked at actors in the choice test. We ran a one-sample t-test within each condition to determine whether the proportion of time that toddlers looked at one actor over the other differed from 50% (i.e., chance). In the Helping Condition, toddlers looked proportionately longer to the Steep Helper during the choice test ($mean_{steep\text{-}choice\%}$

= 56.3%, 95% CI [0.51, 0.61], *SD* = 10.7%, one-sample $t(18) = 2.56$, $p = .019$, $d = 0.58$). In the No Helping Condition, by contrast, toddlers did not look longer to the Steep Pusher in the choice test ($\text{mean}_{\text{steep-choice\%}}$ = 48.4%, 95% CI [0.42.9, 0.53.9], *SD* = 11.7%, one-sample $t(19) = -0.59$, $p = .561$, $d = 0.13$). The proportion of looking to the Steep Helper differed by condition (two-sample $t(36) = 2.18$, $p = .035$, $d = 0.69$).

Next, to examine whether toddlers expected the helpers to help at the steep or the shallow hill in the final events, or were differently interested in these events, we ran a mixed-effects model. We ran a mixed-effects model in which looking time was the dependent variable; event type (Shallow = -0.5, Steep = 0.5), condition (Helping = -0.5, No Helping = 0.5), and their interaction term were included as fixed effects, with a random intercept for participant ID and final event pair (1/2/3) as a random slope. (Because a normal distribution fit these data better than a lognormal distribution did, we did not log transform the looking time data from final events before including them in this model.) As reported in the main text, event type and condition did not significantly predict looking times, and the interaction was not significant (see Table S1).

Because both the choice test and the looking measure in the final events involve toddlers' looking behavior, we next examined whether these looking measures were related to each other in an exploratory analysis. To do so, we first calculated the difference in total looking following final events in which an actor acted on the steep hill vs. the shallow hill. We conducted a beta regression appropriate for the proportion data from the choice test. Specifically, the dependent variable was the proportion of looking at the Steep Actor in the choice test, and the predictors were the difference in total looking following the final events involving the Steep Actor and the Shallow Actor, the Condition (Helping or No Helping), and the interaction between the two. We

found that the difference in looking to the final events involving the Steep Actor vs. the Shallow Actor did not predict toddlers' preference for the Steep Actor (β = -0.05, 95% of β [-0.14, 0.05], *b* = -0.001, *z* = -0.97, *p* = .328), and the interaction was not significant (β = 0.09, 95% of β [-0.04, 0.23], *b* = 0.00, *z* = 1.40, p = .159). Thus, looking in the final events did not appear to be related to looking in the choice test.

Finally, in exploratory analyses, we examined whether there were alternative, low-level reasons for toddlers' looking preferences in the choice test. We found that toddlers did not look significantly differently at actors based on shape or order of presentation (*p*s < .490). However, toddlers looked significantly more at actors on the right ($mean_{right\text{-}choice}$ = 13.56 s, *SD* = 3.91 s) than at actors on the left ($mean_{left\text{-}choice}$ = 10.88 s, *SD* = 3.32 s) (β = 0.70, 95% CI of β [0.28, 1.11], *b* = 2.62, *t*(78) = 3.30, *p* = .001).

To investigate this side preference further, we ran a mixed-effects model in which the dependent variable was time looking at an actor in the choice test; the fixed effects were the actor identity (Shallow = -0.5, Steep = 0.5), condition (Helping = -0.5, No Helping = 0.5), the interaction between identity and condition, and side of helper (left/right); and the random effect was participant ID. Although toddlers looked significantly more to actors on the right than on the left (β = 0.77, 95% CI of β [0.38, 1.16], *b* = 2.95, *t*(78) = 3.87, *p* < .001), the interaction between identity and condition independently and significantly predicted looking and was larger in effect size (β = -1.15, 95% CI of β [-1.93, -0.38], *b* = -4.44, *t*(78) = -2.91, *p* = .004). We therefore concluded that the present interaction is independent of a side preference.

Additional post-hoc pairwise tests, based on this model with actors' sides as a fixed effect and correcting for multiple comparisons, converged with the findings reported in the main text. Specifically, toddlers looked longer to the Steep Helper over the Shallow Helper in the Helping

Condition (β = -0.92, *b* = -3.56, $t(41)$ = -3.14, *p* = .003), but did not distinguish between the Steep and Shallow Pushers in the No Helping Condition (β = 0.23, *b* = 0.88, $t(41)$ = 0.81, *p* = .422).

Experiment 3

The analyses reported in the main text on the choice test were based on analyses of raw preferential looking time. In additional preregistered analyses, we examined what proportion of time toddlers looked at the Weak-Target Helper when they looked at helpers in the choice test. We ran a one-sample *t*-test to determine whether this proportion differed from 50% (i.e., chance). Toddlers looked proportionately longer to the Weak-Target Helper during the choice test ($\text{mean}_{\text{weak-target-choice\%}}$ = 57.1%, 95% CI [0.52, 0.61], *SD* = 12.4%, one-sample $t(31)$ = 2.56, *p* = .002, *d* = 0.56).

Next, to examine whether toddlers expected helpers to help the weaker or the stronger circle in the final events, or were differently interested in these events, we ran an exploratory mixed-effects model. We ran a mixed-effects model in which looking time was the dependent variable and event type (Helped-Strong/Helped-Weak) was included as a fixed effect, with a random intercept for participant ID and final event pair (1/2/3) as a random slope. (Because a normal distribution fit these data better than a lognormal distribution did, we did not log transform the looking time data from final events before including them in this model.) As reported in the main text, event type did not significantly predict toddlers' looking time in final events (β = 0.07, 95% CI of β [-0.16, 0.29], *b* = 0.67, *t* = 0.57, *p* = .569).

We next examined whether the two looking measures (preferential looking in the choice test vs. looking in the final events) were related to each other, as in Experiment 2. We first calculated the difference in total looking following final events in which a helper helped the

Weak Beneficiary vs. the Strong Beneficiary. We conducted a beta regression appropriate for the proportion data from the choice test. Specifically, the dependent variable was the proportion of looking at the Weak-Target Helper in the choice test, and the predictors were the difference in total looking following the final events involving the Weak-Target and the Strong-Target Helpers. We found that the difference in looking to the final events involving the Weak-Target Helper vs. the Strong-Target Helper Actors did not predict toddlers' preference for the Weak-Target Helper ($\beta = 0.00$, 95% of β [-0.07, 0.08], $b = 0.00$, $z = 0.05$, $p = .956$). As in Experiment 2, looking in the final events did not appear to be related to looking in the choice test.

Finally, in exploratory analyses, we examined whether there were alternative, low-level reasons for toddlers' looking preferences in the choice test. We found that toddlers did not look significantly differently at actors based on shape, side, or order of presentation (ps < .184).

Table S1

Mixed-effects Model Output for Toddlers' Looking Times During Final Events in Experiment 2

	β	95% CI of β	*b*	*t*	*df*	*p*
Event Type	-0.03	[-0.21, 0.15]	-0.53	-0.34	118	0.73
Condition	0.06	[-0.27, 0.38]	0.96	0.34	39	0.73
Interaction	0.02	[-0.33, 0.38]	0.42	0.13	113	0.89

SI References

Bates, D., Mächler, M., Bolker, B., & Walker, S. (2015). Fitting Linear Mixed-Effects Models Using lme4. *Journal of Statistical Software*, *67*(1), Article 1. https://doi.org/10.18637/jss.v067.i01

Casstevens, R. M. (2007). jHab: Java habituation software (version 1.0. 2)[computer software]. *Chevy Chase, MD*.

Hamlin, J. K., Hallinan, E. V., & Woodward, A. L. (2008). Do as I do: 7-month-old infants selectively reproduce others' goals. *Developmental Science*, *11*(4), Article 4. https://doi.org/10.1111/j.1467-7687.2008.00694.x

Hamlin, J. K., & Wynn, K. (2011). Young infants prefer prosocial to antisocial others. *Cognitive Development*, *26*(1), Article 1. https://doi.org/10.1016/j.cogdev.2010.09.001

Hamlin, J. K., Wynn, K., & Bloom, P. (2007). Social evaluation by preverbal infants. *Nature*, *450*(7169), Article 7169. https://doi.org/10.1038/nature06288

Hamlin, J. K., Wynn, K., & Bloom, P. (2010). Three-month-olds show a negativity bias in their social evaluations. *Developmental Science*, *13*(6), Article 6. https://doi.org/10.1111/j.1467-7687.2010.00951.x

Jara-Ettinger, J., Tenenbaum, J. B., & Schulz, L. E. (2015). Not so innocent: Toddlers' inferences about costs and culpability. *Psychological Science*, *26*(5), 633–640.

Kuznetsova, A., Brockhoff, P. B., & Christensen, R. H. (2017). lmerTest package: Tests in linear mixed effects models. *Journal of Statistical Software*, *82*, 1–26.

Pinto, J. (1996). *XHAB64*.

Woo, B. M., & Spelke, E. (2023a). Infants and toddlers leverage their understanding of action goals to evaluate agents who help others. *Child Development*.

Woo, B. M., & Spelke, E. (2023b). Toddlers' social evaluations of agents who act on false beliefs. *Developmental Science*. https://doi.org/10.31234/osf.io/eczgp

Woodward, A. L. (1998). Infants selectively encode the goal object of an actor's reach. *Cognition*, *69*(1), Article 1.
